# 

**Published:** 1885-01

**Authors:** 


### INDEX.

PAGE.

Abattoirs ... 246
Abortion in cows, enzöotic ... 25
Absence of Dr. Billings ... 97
Acute parenchymatous nephritis ... 308
Address, introductory ... 1
American veterinarian's impression of the profession in England ... 322
    veterinary college ... 204, 268
Angina paralytic in the horse ... 103
Animal disease, facts as to the stamping out of contagious ... 95
    industry, bureau of ... 197
    slaughtered on account of contagious diseases, remuneration for the loss of ... 92
    diseases to the public health, relation of ... 96
    industry, bureau of ... 98
Annals of surgery ... 112
Anthrax ... 296
    inoclation of Pasteur ... 73
    the etiology of ... 340
Appointment of Dr. Billings ... 198
Arnica, tincture of, accidents produced by ... 214
Art of taming and educating horses ... 109
Autopsies at Central Park Menagerie ... 101, 201, 269, 361
Axis deer, autopsy of ... 269
Azoturia ... 156
    two cases of ... 202

Baboon, autopsy of ... 102
Bacteria cultivation. method of ... 113, 240
Bang, B., tuberculosis of the udder of the milch-cow, and its effect upon the milk ... 143
Bassi, R., some diseases of the ourang-outang ... 270
Beaver, autopsy of ... 101
    tail, innervation of the ... 214
Beyer, H. G., autopsies at Central Park Menagerie ... 201, 269
Biggs, H. W., physiological action of cocaine on the frog ... 128

105010

Billings, F. S., absence of........ 97
appointment of........ 198
bacteria culture........ 240
enzöotic abortion in cows........ 25
State medicine........ 217
Black-leg, inoculation against........ 352
Bollinger, Prof. and Kitt, Theodore, the etiology of anthrax........ 340
Books and pamphlets received........ 112, 208, 290, 376
Borax-methylenblue........ 293
Bouley, Prof........ 294
Brain of monkey........ 209
Bureau of animal industry........ 98, 197
Byrne, Charles J., indigestion in the horse........ 232

Cancer of the penis........ 295
Case department........ 101, 201, 269, 361
of congenital tuberculosis........ 292
Castration of the elephant........ 291
Cats, scarlet fever and measles in dogs and........ 21
Chicago veterinary college........ 281
Chimpanzee, notes on the psychology of the........ 38
Clement, A. W., acute parenchymatous nephritis........ 308
Clinical aspect of some disorders of the gastro-intestinal tract of the horse........ 315
Cocaine, physiological action of........ 128
Colic, its causes........ 364
Contagious animal diseases, facts as to the stamping out of........ 95
diseases, remuneration for the loss of animals slaughtered on account of........ 92
pleuro-pneumonia........ 293
Cooper, Dr. Astley, operations on animals........ 211
Correspondence........ 107, 205, 272, 371
Cows, enzöotic abortion in........ 25
removal of a portion of the teat of........ 102
sugar in the urine of........ 358
surgery........ 214
Crow, autopsy of........ 362

Deer, axis, autopsy of........ 269
Dickerhoff, William, pulmonary emphysema and its relation to heaves in the horse........ 168
Diphtheria, fowl........ 214
Diseases of the ourang-outang........ 270
Dogs and cats, scarlet fever and measles in........ 21
meat as food for man........ 209
Duty on scientific books and instruments........ 354

Editorial department........ 86, 189, 265, 348
Elephant, castration of........ 291
generative organs of........ 291

Emmerich's so-called Naples cholera bacteria........................ 342
Enzöotic abortion in cows........................ 25
Ercolani, eulogy on........................ 297

Facts as to the stamping out contagious animal diseases........................ 95
Food supply and contagious diseases........................ 200
Foot and mouth disease........................ 293
Fowl diphtheria........................ 214

Gangrene........................ 53
Gazelle, autopsy of........................ 270
Generative organs of the elephant........................ 291
Gerth, J., resignation of........................ 370
Glanders, a case of........................ 214
Guzzoni, Melchioire........................ 201

Heard, J. M., human and bovine tuberculosis........................ 333
Heaves, pulmonary emphysema and its relation to, in the horse........................ 168
Hernia, pulmonary, radical cure of........................ 203
History of tuberculosis........................180, 257
Hog-cholera........................ 209
    inoculation of........................ 335
Horse, angina-paralitica in the........................ 103
    art of training and educating........................109
    disorders of the gastro-intestinal tract of the........................ 315
    indigestion in........................ 232
    pericarditis in a........................ 292
    pulmonary empysema and its relation to heaves in the........................ 168
Huidekopfer, R. S., address........................ 1
Human and bovine tuberculosis........................ 333

Iodoform, use of........................ 215
Indigestion in the horse........................ 232
    sulphurated hydrogen as a product of........................ 162
Inoculation against black-leg........................ 352
    anthrax of Pasteur........................ 73
    of hogs, with reference to erysipelas........................ 335
Introductory address........................ 1

Johne, A., history of tuberculosis........................ 180
    Koch's method of bacteria cultivation........................ 113

Koch, Robert........................ 113

Laceration of the flexor metatarse........................ 363
Life-work of Pasteur........................ 62
Lioess, self-mutilation in........................ 210

Mammitis, experience with........................ 331
Marsh, J. W., two cases of azoturia........................ 202

Massachusetts veterinary medical association....................104, 282, 367
McLaughlin, J. A., sulphuretted hydrogen as a product of indigestion.. 162
Measles in dogs and cats.......................................... 21
Medical diploma craze............................................. 266
science, relation of general pathology to......................... 189
Meyer, C. A., experience with mammitis............................ 331
Milch-cow, tuberculosis in the udder of........................... 143
Mitchell, C. P., notes, with commentations on the psychology of the chimpanzee.......................................... 38
Monkey, brain of.................................................. 209
Monstrosity, a.................................................... 213
Montreal veterinary medical association........................... 106

National veterinary association................................... 210
Necessity of an organized inspection of our meat and milk supply...... 348
News and miscellany............................................... 209
New York academy of veterinary science and comparative pathology... 212, 276, 364
polyclinic school of medicine..................................... 268
None too much quarantine.......................................... 350
Notes on the psychology of the chimpanzee......................... 38
veterinary medicine in Great Britain.............................. 19

Obituary..........................................201, 291, 372
Ontario veterinary college.....................................204, 280
Operative veterinary surgery, a text-book of...................... 108
Opossum, the cunning of........................................... 212
Ostrich, autopsy of............................................... 201
Ourang-outang, diseases of........................................ 270

Parrots, causes of death in....................................... 361
Pasteur, anthrax inoculation of................................... 73
life work of...................................................... 62
Penis, cancer of.................................................. 295
Pericarditis in a horse........................................... 292
Personal.......................................................... 209
Peters, A., an American veterinarian's impression of the profession in England............................................ 222
J. C., scarlet fever in dogs and cats.............................. 21
Psychological action of cocaine on the frog....................... 128
Plageman, L. V., colic............................................ 364
laceration of the flexor metatarsi................................ 363
Pleuro-pneumonia, contagious...................................... 293
preventive inoculation of......................................... 294
Proceedings veterinary medical societies ........ 104, 204, 276, 364
Progress of veterinary science.................................214, 291
Prolapse of the rectum in swine................................... 215
Public abattoirs a necessity to health............................ 246
health, relation of the animal diseases to the.................... 96

Pulmonary diseases in wild animals........ 215
emphysema and its relation to heaves in the horse........ 168
hernia, radical cure of........ 203
Pulz, H., consideration of the anthrax inoculation of Pasteur........ 73
Psychology of the chimpanzee, notes on........ 38

Relation of animal disease to the public health........ 96
general pathology to medical science........ 189
Removal of a portion of the teat of a cow........ 102
Remuneration for loss of animals slaughtered........ 92
Reviews........108, 206, 285, 373
Reynolds, E. R., eulogy on Count Ercolani........ 297
Rogers, T. B., azoturia........ 156
disorders of the intestinal tract of the horse........ 315

Scarlet fever and measles in dogs and cats........ 21
School of veterinary medicine of Milan........ 98
Schutz, Prof., inoculation of hogs........ 335
Self-mutilation in a lioness........ 210
Skally, J. M., gangrene........ 53
Spaying bitches........ 265
Stamping out contagious animal diseases........ 95
Starr, M. A., autopsy of tiger........ 361
State medicine........ 217
veterinarians........ 351
Sugar in the urine of cows........ 458
Sulphuretted hydrogen as a product of indigestion........ 162

Tetanus successfully treated........ 102
Thomas, F. S., correspondence........ 205
Tiger, autopsy of........ 361
Toronto veterinary college........ 199
Townsend, N. S., notes on veterinary medicine in Great Britain........ 19
True, F. W., autopsies at Central Park menagerie........ 270
Tuberculosis, congenital, case of........ 292
history of........180, 257
human and bovine........ 333
in the udder of the milch cow........ 143

United States veterinary association........ 358

Velpeau's early career........ 211
Veterinary association, Massachusetts........104, 282
Montreal........ 106
National........ 210
New Jersey........ 369
United States........ 358
college American........204, 268
Chicago........ 281
Ontario........204, 280
Toronto........ 199

department, University, Pa. . . . . . . . . . 100
medical societies, proceedings of. . . . . . . . . .104, 204
medicine in Great Britain, notes on. . . . . . . . . . 19
progress, quarterly report of. . . . . . . . . . 111
questions in the United States. . . . . . . . . . 86
school at Munich . . . . . . . . . . 355
Milan. . . . . . . . . . 98
Vienna. . . . . . . . . . 357
science and art, society of. . . . . . . . . . 204
pathology, academy of. . . . . . . . . . 212
surgery, text-book of. . . . . . . . . . 108
Virchow, R. . . . . . . . . . 253

Walley T., correspondence. . . . . . . . . . 272
Water buck, autopsy of. . . . . . . . . . 270
Wild animals, pulmonary diseases in. . . . . . . . . . 215

Koch and his Methods.—We beg to announce to our readers that the April number of the Journal will contain a portrait of Prof. Koch, as well as an article with the above title from the pen of his friend and co-laborer, Dr. Albert Johne, of Dresden.

### CASE DEPARTMENT.

### BOOKS AND PAMPHLETS RECEIVED.

R. SCUOLA, SUPERIORE DI MEDICINA VETERINARIA DI MILANO. Milan, 1884.

EXPLANATION OF THE PATHOLOGY AND THERAPEUTICS OF THE DISEASES OF THE NERVE-CENTRES. By I. McF. Gaston, M.D. Atlanta, Ga., 1884.

THE DRY TREATMENT OF CHRONIC SUPPURATIVE INFLAMMATION OF THE MIDDLE EAR. By Prof. Charles J. Lundy, M.D. Detroit, 1884.

MURIATE OF COCAINE IN OPHTHALMIC SURGERY. By Charles J. Lundy, M.D. Detroit, 1884.

PHYSICIANS' VISITING LIST FOR 1885. Published by P. Blakiston, Son & Co., Philadelphia.

REPORT OF THE BROOKLYN COMMISSIONER OF HEALTH, December, 1884.

JOHNS HOPKINS UNIVERSITY CIRCULARS.

ANNALS ET BULLETIN DE LA SOCIÉTÉ DE MEDICINE DE GAND.

JOURNALS.—La Clinica Veterinaria, Milano. The Veterinarian, London; The Veterinary Journal, London; Der Zoologische Garten, Frankfort; Schweizerisches Archiv fur Thierheilkunde, Zurich; Archiv fur Wissenschaftliche und Praktische Thierheilkunde, Berlin; Wochenschrift fur Thierheilkunde und Viehzucht, Augsburg; Tidskrift for Veterarin-Medicin, Stockholm; Journal de la Société contre L'Abus du Tabac, Paris; Giornale di Anatomia, Fisiologia e Patologia, degli Animali, Pisa; La Cronica Medica, Valencia; Kansas City Review of Science and Industry; The College and Clinical Record, Philadelphia; New England Medical Monthly, Sandy Hook; Nashville Journal of Medicine and Surgery; The Southern Clinic, Richmond; The Western Medical Reporter, Chicago; Journal of Cutaneous and Venereal Diseases, New York; Denver Medical Times; The St. Joseph Medical Herald; The Weekly Medical Review, St. Louis; The Blacksmith and Wheelwright, New York; National Live Stock Journal, Chicago; The Breeder's Gazette, Chicago; Cultivator and Country Gentleman, Albany; Weekly Drover's Journal, Chicago; The Chicago Tribune; Horseshoer and Hardware Journal, Chicago; The Polyclinic, Philadelphia; Medical World, Philadelphia; American Veterinary Review, New York; Atlantic Journal of Medicine, Richmond; Indiana Farmer, Indianapolis; Texas Courier Record, Fort Worth; The Jersey Bulletin, Indianapolis; American Journal of Ophthalmology, St. Louis; Pacific Medical and Surgical Journal and Western Lancet, San Francisco; New York Medical Journal; The Quarterly Journal of Veterinary Science in India; Hearth and Home, Washington; American Liveryman and Horseowner, Chicago; American Psychological Journal, Philadelphia.

